# Improved Grain Boundary Reconstruction Method Based on Channel Attention Mechanism

**DOI:** 10.3390/ma18020253

**Published:** 2025-01-08

**Authors:** Xianyin Duan, Yang Chen, Xianbao Duan, Zhijun Rong, Wunan Nie, Jinwei Gao

**Affiliations:** 1 Key Laboratory of Metallurgical Equipment and Control Technology, Ministry of Education, Wuhan University of Science and Technology, Wuhan 430081, China; xyduan@wust.edu.cn (X.D.); wustcy@163.com (Y.C.); 2 Hubei Key Laboratory of Plasma Chemistry and Advanced Materials, School of Materials Science and Engineering, Wuhan Institute of Technology, Wuhan 430205, China; nange111111@163.com (W.N.); gaojinwei131774@163.com (J.G.)

**Keywords:** grain boundary reconstruction, channel attention mechanism, generative adversarial network (GAN), mode collapse, grain size, machine learning, artificial intelligence

## Abstract

The grain size of metal materials has a significant impact on their macroscopic properties. However, original metallographic images often suffer from issues such as substantial noise, missing grain boundaries, low contrast, and blurred edges. These challenges hinder the accurate extraction of complete grain boundaries, limiting the precision of grain size measurement and material performance prediction. Therefore, effectively reconstructing incomplete grain boundaries is particularly crucial. This paper proposes a grain boundary reconstruction and grain size measurement method based on an improved channel attention mechanism. A generative adversarial network (GAN) serves as the backbone, with a custom-designed channel attention module embedded in the generator. Combined with a global context attention mechanism, the method captures the global contextual information of the image, enhancing the network’s semantic understanding and reconstruction accuracy for regions with missing grain boundaries. During the image reconstruction process, the method effectively leverages long-range feature correlations within the image, significantly improving network performance. To address the Mode Collapse observed during experiments, the loss function is optimized using Focal Loss, balancing the ratio of positive and negative samples and improving network robustness. Compared with other attention modules, the improved channel attention module significantly enhances the performance of the generative network. Experimental results demonstrate that the generative network based on this module outperforms comparable modules in terms of MIoU (86.25%), Accuracy (95.06%), and Precision (86.54%). The grain boundary reconstruction method based on the improved channel attention mechanism not only effectively improves the accuracy of grain boundary reconstruction but also significantly enhances the generalization ability of the network. This provides reliable technical support for the characterization of the microstructure and the performance prediction of metal materials.

## 1. Introduction

Metal materials are widely used in critical fields such as energy and power, transportation, and national defense, serving as strategic foundational materials that support national economic development. The grain size of metal materials has a significant impact on many of their properties, reflecting not only the microstructural characteristics of the material but also directly influencing its macroscopic properties such as yield strength, toughness, wear resistance, and corrosion resistance [1,2,3,4,5]. Accurately measuring grain size is crucial to ensuring that the material’s performance meets the specific requirements of its application. However, the complex preparation and acquisition processes of metallographic images often result in substantial noise and missing grain boundary regions in the images, posing challenges for the accurate identification and measurement of grain size.

Grain boundary reconstruction is an effective solution for achieving precise grain size measurement. It supports microstructural characterization and the macroscopic performance prediction of materials, advancing the development of material research and applications. Metallographic images exhibit intensity differences between grain boundary pixels and interior pixels, which traditional image-processing algorithms typically leverage for segmentation. However, due to the influence of factors such as lighting and preparation techniques during the image preparation process, metallographic images often have low contrast, as well as issues like significant noise and scratches.

Currently, grain size detection mainly relies on manual annotation to analyze metallographic images and obtain measurement results. This method is inefficient and heavily depends on the expertise and skill level of professionals, making it difficult to ensure the consistency, reproducibility, and interpretability of the analysis. Commercial metallographic analyzers equipped with dedicated image-processing software can improve efficiency but struggle to fully identify grain boundaries in images with unclear boundaries or low contrast. The extracted grain boundary maps often contain missing regions, and the software lacks the capability to repair these missing areas, which compromises the accuracy of grain size calculations. Therefore, repairing and reconstructing missing grain boundaries in metallographic images is essential for improving the accuracy of grain size measurement.

Extensive research has been conducted to address the problem of grain boundary extraction. Subir Gupta et al. [6] employed the Otsu operator for image segmentation, a clustering-based image thresholding technique. However, the significant intensity variation between the grain interior and the grain boundaries notably affects the segmentation results. To address this issue, Yuhan Wang [7] proposed an edge detection algorithm based on fuzzy logic, while Siddhartha Banerjee et al. [8] adopted an edge detection method based on the Canny algorithm. When the grain interiors exhibit uniform intensity distribution and the boundary regions demonstrate significant intensity changes, edge detection methods can effectively identify grain boundaries. However, in practical applications, if the boundary features are not prominent, discontinuities in grain boundaries may occur. Additionally, while the Canny edge detection method yields satisfactory results for grain boundary extraction in low-resolution metallographic images, its performance deteriorates in high-resolution images. Lucijano Berus et al. [9] utilized a local Laplacian approach, achieving better results for grain boundary extraction even in high-resolution metallographic images. Nevertheless, edge detection methods often result in double edges for thicker boundaries, which compromises the accuracy of grain boundary extraction. Overall, edge detection methods suffer from significant drawbacks, such as generating incomplete grain boundaries or redundant edges, which severely limit the accuracy of grain size measurement.

In recent years, deep learning algorithms have been increasingly applied to visual tasks in the field of metallic materials [10,11,12]. Compared to traditional image-processing algorithms, deep learning models exhibit stronger capabilities for capturing high-level semantic information and improved noise robustness. The U-Net network [13,14], due to its high accuracy in image segmentation, has become an essential tool in the domain of grain boundary extraction. To further enhance segmentation accuracy, many researchers have optimized the U-Net architecture. Matthew J. Patrick [15] and Peng Shi [16] achieved promising results in metallographic image segmentation tasks by improving the U-Net network. However, they were still unable to extract complete grain boundaries. Mingchun Li et al. [17] proposed a two-stage network to obtain more complete grain boundaries. In the first stage, multi-task learning was used to perform grain boundary segmentation, while in the second stage, a generative adversarial network (GAN) was employed to optimize the results of the first stage, ultimately achieving a more complete grain boundary extraction.

Despite the superior performance of deep learning models, their dependence on large-scale training datasets has become a limiting factor. In metallography, the complexity of image preparation and the difficulty in acquiring substantial data during material development often result in insufficient training data, hindering the full potential of these models. To address this issue, Peter Warren et al. [18] proposed using image generation techniques to artificially synthesize metallographic images. This method effectively augments dataset size and enhances image realism by introducing noise and scratches into synthetic images. Although this approach improves network performance to some extent, it fails to resolve the issue of missing grain boundaries, which compromises the accuracy of grain size rating.

Reconstructing missing grain boundaries is thus a critical step for accurately assessing material performance. Developing effective methods for grain boundary reconstruction is crucial for improving the precision of microstructural analysis in materials science. Grain boundary reconstruction aims to restore damaged regions of grain boundary images with realistic textures and structures. Given the exceptional performance of generative adversarial networks (GANs) in image generation [19,20] and the efficacy of attention mechanisms in enhancing feature extraction [21,22], incorporating attention mechanisms can further improve network performance.

In this study, GANs are applied as the backbone network for image reconstruction, and an improved channel attention mechanism-based model is proposed for grain boundary reconstruction. A custom-designed channel attention module is embedded in the generator, combined with a global context attention mechanism, to capture global contextual information in feature maps. This enhances the network’s semantic understanding and reconstruction capabilities for missing grain boundary regions. During experiments, Mode Collapse was observed when sparse line inputs were used, leading to significantly decreased network performance. To mitigate this issue, the loss function was optimized by introducing Focal Loss [23] to balance the ratio of positive and negative samples, thereby improving reconstruction outcomes.

## 2. Grain Boundary Reconstruction Model

**Figure 1:**
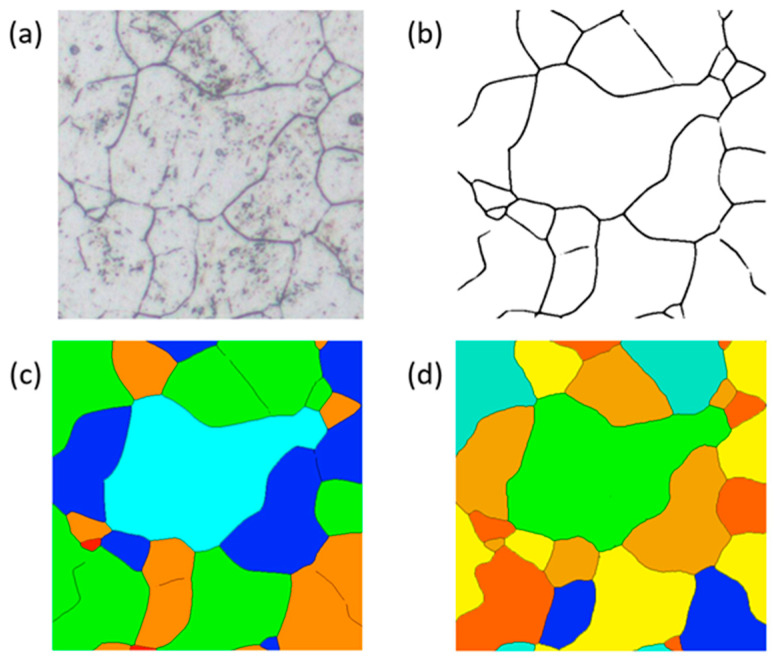
Grain boundary extraction and grain labeling. (**a**) Original metallographic image. (**b**) Grain boundary map. (**c**) Grain boundary non-reconstructed grain labeling map. (**d**) Grain boundary reconstructed grain labeling map. (The colors in the figure are only for distinguishing different grains to show the changes in grains before and after reconstruction, and have no other meaning).

In metallographic analysis, accurate grain size calculation is vital for evaluating material performance, as it directly correlates with the mechanical properties and service behavior of metals. However, the complexity of metallographic image preparation often results in significant noise, scratches, and broken or blurred regions in images (as shown in Figure 1a). These defects pose substantial challenges for extracting grain boundaries and calculating grain size. Boundaries extracted using traditional image-processing methods (Figure 1b) often exhibit missing or incomplete regions, rendering them inadequate for material performance analysis.

The process of extracting and annotating grain boundaries in metallographic analysis is shown in Figure 1. Based on the original metallographic image shown in Figure 1a, a U-Net network is used to extract the grain boundary map (Figure 1b). Grain annotations without and with reconstructed boundaries are shown in Figure 1c,d. A comparison between Figure 1c,d reveals significant differences in grain size and count, which directly influence the accuracy of grain size calculation and subsequent material performance evaluation.

To address the issue of missing grain boundaries and enhanced grain size calculation accuracy, a GAN-based grain boundary reconstruction method is proposed. Considering the complexity of this task, a custom-designed channel attention module (MCANet) is introduced into the generator of the GAN backbone network. This module effectively models global contextual relationships, enhancing the network’s ability to capture features specific to grain boundary regions.

**Figure 2:**
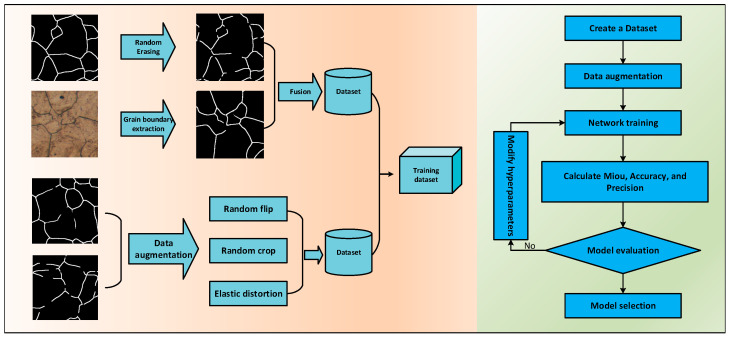
Workflow diagram.

Additionally, to tackle the imbalance between foreground and background pixels in grain boundary images, which often leads to Mode Collapse during training, the loss function of the conditional GAN [24] was improved by incorporating Focal Loss. This modification not only balances the weight distribution between foreground and background during training but also produces more visually plausible reconstructed boundaries. The workflow diagram [25] is shown in Figure 2.

### 2.1. Improved Channel Attention Mechanism

Attention mechanisms can effectively model long-range dependencies, making them widely applicable in image generation and restoration tasks. Studies [26,27] suggest that capturing pixel-wise dependencies is critical for understanding the global context in visual scenes. Therefore, a new channel attention module is designed, incorporating global context attention to modeling these relationships. Unlike convolutional neural networks that rely on repeated convolution operations for dependency modeling, the proposed module inherently establishes global dependencies without requiring deep network layers. This design enhances computational efficiency and facilitates optimization through residual connections.

**Figure 3:**
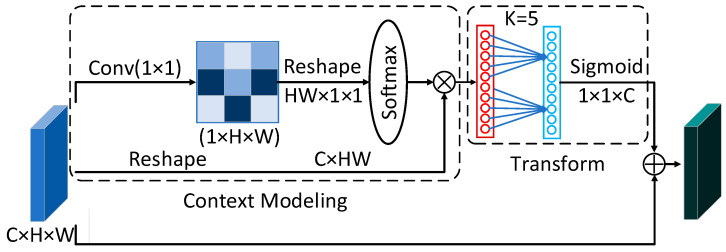
Improved channel attention module.

A schematic of the proposed channel attention module is shown in Figure 3. The module begins by computing inter-feature relationships using 1 × 1 convolution and a Softmax function to generate attention maps. These maps are used to calculate weighted sums of features at all positions, completing feature aggregation and generating the output. To compute channel-wise attention effectively, the module employs global context attention for spatial information aggregation, avoiding the information loss often associated with average or max pooling. This approach strengthens the module’s capacity for long-range dependency modeling and enhances its performance.

Additionally, studies have shown that appropriate cross-channel interactions can significantly reduce model complexity while maintaining network performance [28]. The module designed in this paper calculates the cross-channel interaction process and generates corresponding channel weights through one-dimensional convolution operations. In the Transform submodule of the designed module, K is adaptively determined through the mapping of the channel dimension *C*. The specific formulation of the channel attention module is as follows

(1) $$z_{i}=F(x_{i},\delta (\sum_{j=1}^{N_{p}}\alpha_{j}x_{j}))$$

where $\sum_{j=1}^{N_{p}}\alpha_{j}x_{j}$ represents the context modeling module, which combines features from all positions through weighting and uses the weight *α_j_* to obtain global context features. *δ*(∙) denotes the transformation that captures relevant features, and *F*( · , · ) represents the fusion function used to aggregate global context features across all positions.

To validate the effectiveness of the proposed channel attention module, this study employs a generative adversarial network (GAN) as the backbone framework, integrating the U-Net architecture for tasks with small datasets. The U-Net structure, comprising a contraction path and a symmetric expansion path, uses skip connections to transfer features directly from the encoder to the decoder, thereby enhancing detail retention. Given its simplicity and expandability, the proposed channel attention module is embedded in the contraction path of the U-Net to improve feature capturing and long-range dependency modeling, enhancing GAN performance in image inpainting tasks.

### 2.2. Improved Loss Function

This study utilizes a GAN as the backbone framework for grain boundary reconstruction, transforming input grain boundary images into target images. In conditional GANs (cGANs) [24], both L1 Loss and GAN Loss are commonly used as loss functions. However, due to the significant imbalance between foreground and background pixels in grain boundary images, the training process encountered Mode Collapse, rendering the model incapable of proper learning.

**Figure 4:**
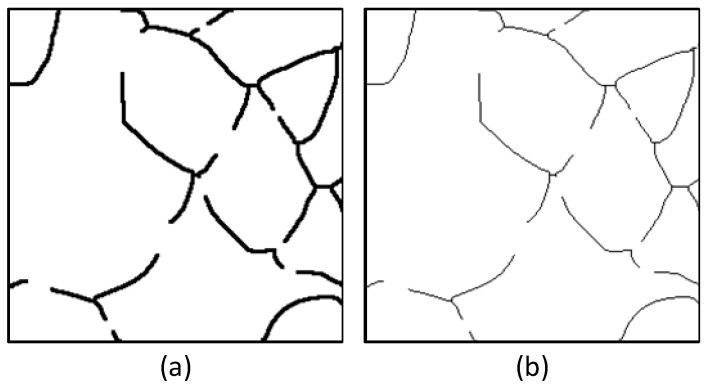
Data input and skeletonized image. (**a**) Original training image; (**b**) skeletonized image.

As illustrated in Figure 4, the training images and their corresponding skeletonized images show that the foreground pixels are significantly fewer than the background pixels. Additionally, a comparison of the dataset used in this study with the official cGAN dataset reveals a marked class imbalance in the input images. This imbalance leads the model to favor background pixels during training, adversely affecting performance.

To validate this reasoning, two comparative experiments with varying amounts of original training and skeletonized images were conducted:

Experiment 1: Training on skeletonized grain boundary images reduced to single-pixel boundaries. The model exhibited Mode Collapse, failing to train properly.

Experiment 2: Training on skeletonized images processed using morphological dilation. In this case, training proceeded successfully, yielding valid results.

These experiments confirm that the severe imbalance between foreground and background pixels is the primary cause of training failure. This issue, fundamentally a positive–negative sample imbalance, is a classic challenge in image-processing tasks.

To address this problem, this study incorporates the Focal Loss function into the cGAN loss framework. The proposed loss function combines Focal Loss, GAN Loss, and L1 Loss into a unified weighted loss function termed GANLoss + L1 Loss + FL Loss. Focal Loss dynamically adjusts the contribution of positive and negative samples, significantly improving model performance.

The Focal Loss is defined as follows:

$$FL=-\frac{1}{N}\sum_{i=1}^{N}\left(\alpha y_{i}{\left(1-p_{i}\right)}^{\gamma }\log p_{i}+(1-\alpha )p_{i}^{\gamma }(1-y_{i})\log(1-p_{i}\right))$$

where *α* serves as a scaling factor to reduce the relative loss of well-classified samples by ${\left(1-p_{i}\right)}^{\gamma }$, allowing the model to focus more on misclassified samples. This improves network accuracy in scenarios with a high number of simple samples.

For the cGAN, the loss function is expressed as:

$$L_{cGAN}(G,D)=E_{x,y\sim p_{data}(x,y)}[\log D(x,y)]+E_{x\sim p_{data}(x),z∼p_{z}(z)}[\log(1-D(x,G(x,z)))]$$

By jointly employing L1 Loss and cGAN Loss during image reconstruction, the generated images exhibit finer details. The discriminator remains unchanged, while the generator not only deceives the discriminator but also generates images closer to the ground truth under the L1 Loss constraint. The L1 Loss is defined as:

$$L_{L1}(G)=E_{x,y\sim p_{data}(x,y),z\sim p_{z}[||y-G(x,z)|{|}_{1}]}$$

The proposed loss function combines Focal Loss, L1 Loss, and cGAN Loss as follows:

$$L\mathrm{oss}=FL+L_{cGAN}(G,D)+\lambda L_{L1}(G)$$

where *λ* is a balancing coefficient set to 100 [24] to achieve optimal results on the validation set.

Considering the significant proportion of negative samples, *α* is set to 0.25 [23] to balance the positive and negative samples. After incorporating the improved loss function, the model no longer experienced Mode Collapse during training and generated visually plausible grain boundaries.

### 2.3. Dataset Preparation and Training

To evaluate the effectiveness of the proposed channel attention module, a grain boundary image dataset was constructed. In image inpainting tasks, masking techniques are often used to obscure certain regions of an image to generate training samples [29,30]. Similarly, this study partially erases grain boundaries in labeled images to create a training dataset.

**Figure 5:**
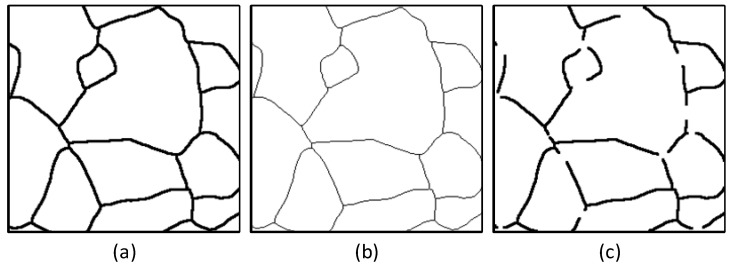
Grain boundary dataset creation process. (**a**) Original label image; (**b**) skeletonized label image; and (**c**) input training image.

The dataset preparation process is illustrated in Figure 5.

(1) Skeletonize the labeled images into single-pixel images (Figure 5b).

(2) Randomly select 9–16 points in the skeletonized images and erase 5–10 pixels around these points.

(3) Restore the erased images into training samples using morphological dilation (Figure 5c).

This random erasing operation simulates potential random missing of grain boundaries during the grain boundary prediction process, improving the prediction accuracy of the network and enhancing the robustness of the network.

A total of 20,000 training samples were generated from 176 images with a resolution of 2580 × 1944 using overlapping cropping. During each training epoch, data augmentation techniques such as scaling, rotation, and elastic deformation were applied to enhance sample diversity, thereby improving the network’s robustness and generalization capabilities.

The model was implemented using PyTorch (2.0.0+cu118), adhering to standard methods described in the literature [24]. Training alternate gradient descent optimizations on the discriminator D and generator G, with network parameters optimized using the stochastic gradient descent (SGD) algorithm.

Key training configurations include:

(1) Batch size: 1 (optimal prediction performance).

(2) Convolution operations: Zero Padding to maintain input-output resolution consistency.

(3) Overfitting mitigation: Batch Normalization, Dropout, and real-time data augmentation.

(4) Learning rate: Initially set to 0.0002, with linear decay after 100 epochs until 200 epochs. The formula for linear decay is

(2) $$l_{r}=l_{r}\times [1-\frac{\max(0,epoch-99)}{101}]$$

where epoch is the number of training rounds, set to 200.

Three widely used metrics—Mean Intersection over Union (MIoU), Accuracy, and Precision—were employed to comprehensively evaluate the network’s performance on grain boundary prediction tasks. These metrics provide a holistic view of the model’s effectiveness and reliability.

## 3. Results and Discussion

To validate the effectiveness of the improved grain boundary reconstruction model, this section presents a series of ablation experiments. These experiments assess the contributions of the channel attention mechanism and the enhanced loss function to the performance of grain boundary reconstruction. The evaluation focuses on three quantitative metrics: Accuracy, Precision, and MIoU (Mean Intersection over Union).

### 3.1. Performance Analysis of the Channel Attention Module

The improved channel attention module integrates global context attention to capture global contextual information, enabling the effective modeling of long-range dependencies and enhancing the overall network performance. To demonstrate the superiority of this module, its reconstruction results are compared with those of SENet and ECANet, as shown in Table 1.

The results in Table 1 reveal that the proposed channel attention module (MCANet) outperforms SENet and ECANet across all three key metrics: MIoU, Accuracy, and Precision. Specifically, compared to ECANet, MCANet improves MIoU by 4.96%, Accuracy by 2.69%, and Precision by 1.98%. This demonstrates the significant enhancement in reconstruction performance achieved by the proposed approach.

The superior performance of the proposed module across MIoU, Accuracy, and Precision highlights its ability to improve the accuracy of reconstructed grain boundary images, making the results closer to ground truth labels. These improvements are particularly significant for restoring missing regions into grain boundaries, confirming the module’s effectiveness and practical value.

**Figure 6:**
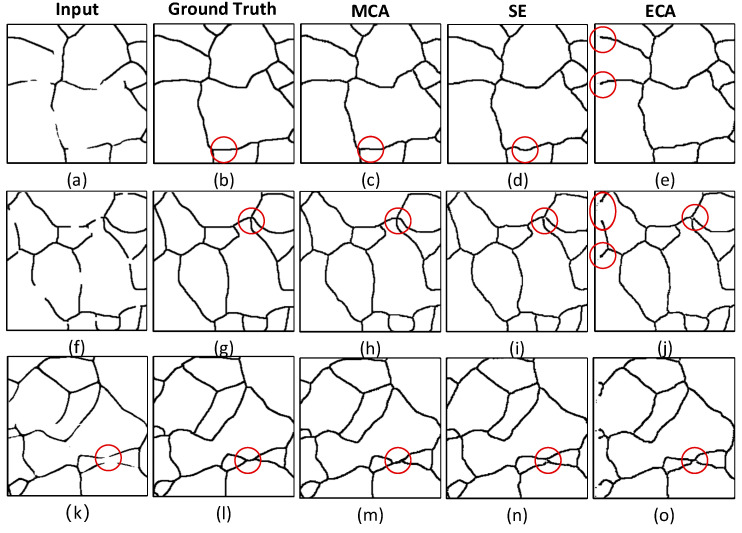
Grain boundary reconstruction results of different attention modules. (**a**,**f**,**k**) Input images; (**b**,**g**,**l**) GroundTruth; (**c**,**h**,**m**) repair images using MCANet; (**d**,**i**,**n**) repair images using SENet; (**e**,**j**,**o**) repair images using ECANet. (The red circles represent the defect area to make the target attention area more prominent).

The visual comparison of reconstruction results using different attention modules is illustrated in Figure 6. Focusing on the regions highlighted by red circles in Figure 6b–e, it is evident that the results generated by the proposed MCANet module (Figure 6c) are closest to the ground truth (Figure 6b). In contrast, the reconstruction by SENet (Figure 6d) shows some degree of distortion, while ECANet (Figure 6e) fails to reconstruct the boundary effectively in certain areas, displaying notable deficiencies.

Further analysis of the highlighted regions in Figure 6g–j reveals issues with SENet (Figure 6i,n) and ECANet (Figure 6j,o) at multi-line intersection points, where the reconstructed boundaries exhibit breaks and fail to form closed structures. By comparison, the proposed MCANet module (Figure 6h,m) successfully reconstructs complete and closed intersections, clearly outperforming the other modules.

In summary, the proposed channel attention module demonstrates significant advantages in capturing global context information and enhancing semantic consistency. It exhibits robust reconstruction capabilities, especially for complex structures such as intersection points, while effectively minimizing distortion and deformation. These results underscore the module’s superior performance and practical applicability.

### 3.2. Performance Analysis of the Loss Function

To address the imbalance between positive and negative samples in the image, this study introduces an improved loss function. While the baseline cGAN network employs GAN Loss and L1 Loss, the proposed approach incorporates Focal Loss (FL Loss) to balance sample contributions and enhance model performance. The improved loss function is a weighted combination of GAN Loss, L1 Loss, and Focal Loss (GANLoss + L1 Loss + FL Loss). The effectiveness of this improvement is verified through ablation experiments, with results summarized in Table 2.

The experiments reveal that, with Focal Loss parameters *α* = 0.25 and *γ* = 2, the model achieves significant performance gains: MIoU reaches 85.19%, Accuracy reaches 94.21%, and Precision reaches 86.07%, representing improvements of 4.17%, 2.07%, and 4.89%, respectively, compared to the baseline loss function. By dynamically adjusting the influence of Focal Loss, the relative weight of simple samples is significantly reduced, while that of hard-to-classify samples is increased. This mechanism enhances the model’s ability to learn from complex samples, improving its classification performance for challenging cases.

**Figure 7:**
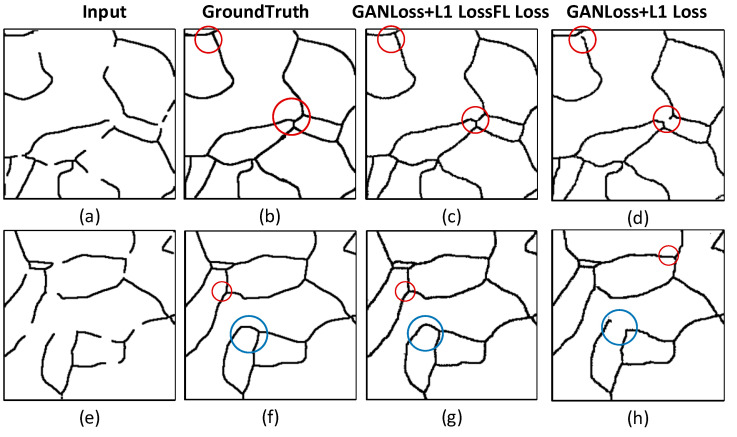
Grain boundary reconstruction using the same generative network and different loss functions. (**a**,**e**) Input image; (**b**,**f**) GroundTruth; (**c**,**g**) repair images using modified loss function; (**d**,**h**) repair images using original loss function. (The red and blue circles represent the defect area to make the target attention area more prominent).

Further analysis of the grain boundary reconstruction task was conducted based on ablation experiments. The reconstruction results are shown in Figure 7, comparing models with the improved loss function (GANLoss + L1 Loss + FL Loss) and the baseline loss function (GANLoss + L1 Loss) in two representative scenarios:

(1) Missing intersections at multi-line junctions (Figure 7a).

(2) Gaps near breakpoints with no clearly connectable endpoints (Figure 7e).

Both scenarios employed the same generative network.

In Figure 7c,d, the regions marked by red circles demonstrate that the improved loss function enables the model to generate structurally complete and closed grain boundaries (Figure 7c), significantly outperforming the baseline loss function, which results in noticeable long-segment gaps (Figure 7d). Similarly, in Figure 7g,h, the improved loss function further enhances the network’s understanding and reasoning capabilities, allowing it to effectively reconstruct missing areas near breakpoints (Figure 7g). The baseline model, however, still exhibits short-segment gaps in these areas (Figure 7h).

Notably, in the large blue circle area, the baseline model fails to address gaps near breakpoints with no clearly connectable endpoints. In contrast, the improved model, by weighing the contribution of hard-to-classify samples, successfully completes the reconstruction. This demonstrates that the introduction of Focal Loss significantly enhances the network’s adaptability to complex scenarios, effectively addressing the limitations of standard loss functions in grain boundary reconstruction tasks.

### 3.3. Validation of Grain Boundary Reconstruction Effectiveness by Grain Size Grading

To evaluate the effectiveness of the proposed grain boundary reconstruction model for accurately measuring grain size, this section conducts grain size grading, a statistical analysis of grains in different grades, and comparisons of grain size distributions before and after reconstruction.

In metallographic image analysis, grains are regions surrounded by grain boundaries, and their sizes are typically calculated using the Comparison Method, Intersection Method, and Area Method. The Comparison Method assesses grain size by comparing the grain boundary images with a set of standard grading images. This method achieves high accuracy only when the grain morphology closely matches the standard grading images, making it sensitive to image quality. Consequently, it may fail to provide reliable results when analyzing complex metallographic images due to the lack of suitable reference standards.

The Intersection Method determines grain size by counting the number of intersections between grain boundaries and a given measurement line or grid. The Area Method calculates grain size grades by counting the number of grains within a specific grid area. Both the Intersection and Area Methods exhibit similar levels of accuracy; however, the Area Method offers a more intuitive understanding of the microstructural distribution of metals by counting grains of different grades. The precision of this method is a function of grain size and can achieve a measurement accuracy of ±0.25 grade through reasonable counting. Therefore, in this study, grain size grade *G* is calculated using the Area Method [31], expressed as

*G* = 3.322lg*N_A_* − 2.954 (3)

where *N_A_* represents the number of grains per square millimeter, calculated as

(4) $$N_{A}=\frac{M^{2}-N}{A}$$

where *M* denotes the magnification, *A* is the area of the measured region, and *N* represents the total number of grains within the measurement grid, expressed as

(5) $$N=N_{\mathrm{inner}}+\frac{1}{2}N_{\mathrm{intersect}}+1$$

where *N*_inner_ indicates the number of grains fully contained within the grid and *N*_intersect_ is the number of grains intersected by the grid.

**Figure 8:**
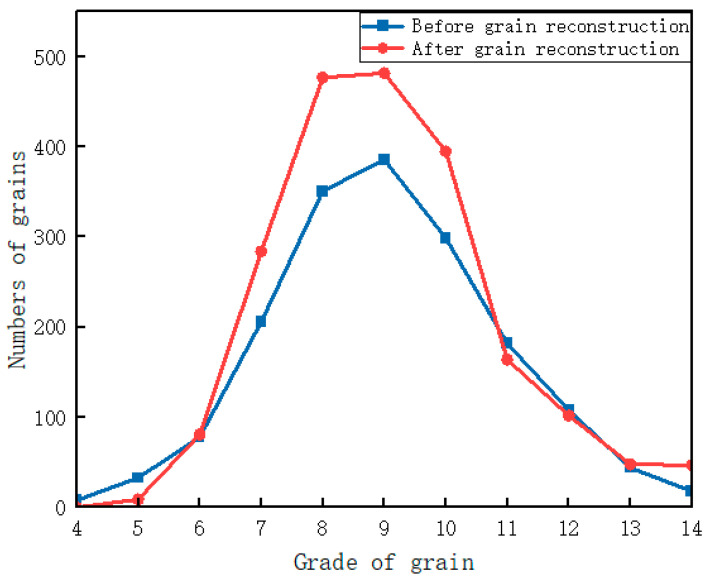
Grain count statistics at different grain levels.

In the experiment, a set of 10 grain sample images was selected for measurement under a magnification of 500×. Using Equation (2), the grain size grade was calculated, and the numbers of grains at different grades were statistically analyzed. The results are presented in Figure 8. The figure shows that the number of grains across all grade changes, demonstrating that the proposed model effectively reconstructs grain boundaries across different grain size grades. This validates the model’s capability to adapt to grains of varying grades during reconstruction.

For grades 7 to 10, their numbers increased significantly. This phenomenon is closely related to the grain characteristics of the material: grains in this range are concentrated, and the reconstruction process supplements previously uncounted grains due to missing boundaries, leading to a noticeable increase in their numbers.

Conversely, the number of grains in some grades, such as grades 4, 5, and 11, decreased. This occurs because grain boundary reconstruction restores the actual sizes of grains, resulting in their reassignment to more accurate grades. Such reassignments typically occur when missing boundaries are repaired, leading to the reclassification of grains previously misjudged as lower grades into higher grades. Therefore, although some grades show a decline in grain numbers, this reflects improved measurement accuracy.

Additionally, the number of grade 14 grains increased from 18 to 47, representing a growth rate of 161%. As the highest-grade grains in this material, their significant recovery after grain boundary reconstruction indicates that a large proportion of high-grade grains were initially misclassified due to missing boundaries. This misclassification likely stems from the susceptibility of the smallest grains to image distortions during sample preparation and imaging.

In summary, grain boundary reconstruction generally increases the number of grains in medium and high grades, particularly in the highest grade, resulting in a more uniform distribution of grains in the region. Furthermore, grain size measurements become more accurate, and the distribution of grain grades is more objective. This facilitates a comprehensive understanding of the material’s microstructural characteristics, providing strong support for more accurate evaluations of material properties.

According to the GB/T 6394-2017 standard [31], the grain size grade *G* can be further calculated by statistically analyzing the number of grains in each grade. The study shows that the grain size grades before and after grain boundary reconstruction are 7.740 and 8.003, respectively (Table 3). This indicates that the grain size grade *G* significantly improves after reconstruction. The results suggest that grain boundary reconstruction effectively reduces errors in grain size statistics caused by incomplete grain boundaries, thereby enhancing the accuracy of grain size calculations and improving the reliability of measurement results.

## 4. Conclusions

This paper proposes an improved channel attention module that incorporates a global context attention mechanism to capture global context information, enhancing the network’s understanding of image semantic information. By embedding this module into the generator of a generative adversarial network, the network’s performance is significantly improved. The proposed method does not require specifying the inpainting region of an image using masks; instead, it automatically learns the structural information of the image, enabling accurate detection and filling of missing grain boundaries. Additionally, the generated grain boundaries maintain consistency in thickness and curvature with natural grain boundaries.

Regarding the issue of the imbalanced proportion of positive and negative samples in metallographic images, this study introduces the Focal Loss function to optimize the original loss function of the generative network. Ablation experiments demonstrate that the introduction of Focal Loss effectively mitigates the imbalance problem, resulting in improvements in MIoU, Accuracy, and Precision by 4.17%, 2.07%, and 4.89%, respectively. In comparisons of ablation experiments across different modules, the proposed channel attention module outperforms the SENet and ECANet modules, achieving MIoU, Accuracy, and Precision of 86.25%, 95.06%, and 86.54%, respectively.

By comparing grain sizes before and after grain boundary reconstruction, the proposed method increases the grain size grade from 7.740 to 8.003, indicating that the reconstructed regions are more uniform. This improvement enhances the accuracy of grain size measurement, offering significant implications for microstructural characterization and macroscopic performance prediction. This work is expected to advance the application of computer vision technology in materials science.

Although the model enhances the network’s ability to understand grain boundary images by modeling long-range dependencies. Additionally, the integration of the Focal Loss function addresses the imbalance between positive and negative samples in the image, improving the network’s repair capabilities. However, the model still faces certain limitations when dealing with excessively long grain boundary discontinuities and noise interference. We believe there are two improvement strategies for addressing these issues. First, the dataset should be expanded, particularly by incorporating more data samples containing complex metallographic images. These datasets should include more instances with high-noise backgrounds and ultra-long discontinuous grain boundaries to improve the model’s generalization ability in atypical scenarios. Second, to overcome computational limitations, higher-performance hardware support should be introduced. This would allow the input of higher-resolution images containing more learnable features for the network (the current input resolution is 256 × 256). For instance, utilizing high-performance GPUs and other computational resources can significantly enhance the model’s training speed and inference efficiency, enabling it to produce more accurate repair results when handling long-distance discontinuities and complex scenarios.

## Figures and Tables

**Table 1:** Comparison of model performance evaluation metrics for different attention modules. (√ denotes the corresponding module is added).

BaseNet	SENet	ECANet	MCANet	MIoU	Accuracy	Precision
U-Net256	√			85.31%	94.96%	84.88%
U-Net256		√		81.29%	92.37%	84.56%
U-Net256			√	86.25%	95.06%	86.54%

**Table 2:** Comparison of model performance metrics for loss functions before and after improvement.

Loss Function	MIoU	Accuracy	Precision
L1 + GAN Loss	81.02%	92.14%	81.18%
L1 + GAN Loss + FL Loss	85.19%	94.21%	86.07%

**Table 3:** Comparison of grain size level results by experiment.

Grain Image	Grain Size Level *G*
Before Grain Boundary Reconstruction	7.740
After Grain Boundary Reconstruction	8.003

## Data Availability

The original contributions presented in this study are included in the article. Further inquiries can be directed to the corresponding authors.
